# Peroral Endoscopic Myotomy for Esophageal Achalasia by HybridKnife: A Case Report

**DOI:** 10.1155/2012/325479

**Published:** 2012-07-31

**Authors:** Ping-Hong Zhou, Ming-Yan Cai, Li-Qing Yao, Yun-Shi Zhong, Zhong Ren, Mei-Dong Xu, Xin-Yu Qin

**Affiliations:** ^1^Endoscopy Center and Endoscopy Research Institute, Zhongshan Hospital, Fudan University, Shanghai 200032, China; ^2^Department of General Surgery, Zhongshan Hospital, Fudan University, Shanghai 200032, China

## Abstract

This paper presented a case of esophageal achalasia treated by peroral endoscopic myotomy with HybridKnife and discuss the feasibility and the possible advantages of using it.

## 1. Case Report 

A 23-year-old lady presented to our hospital complaining of dysphagia for more than one year. Radiographic and manometric evidence supported the diagnosis of achalasia. After obtaining the institutional review board approval and informed consent, peroral endoscopic myotomy (POEM) was performed by an experienced endoscopist (Dr. Zhou, P.H.) with HybridKnife (T-type, ERBE, Germany).

**Figure 1:**
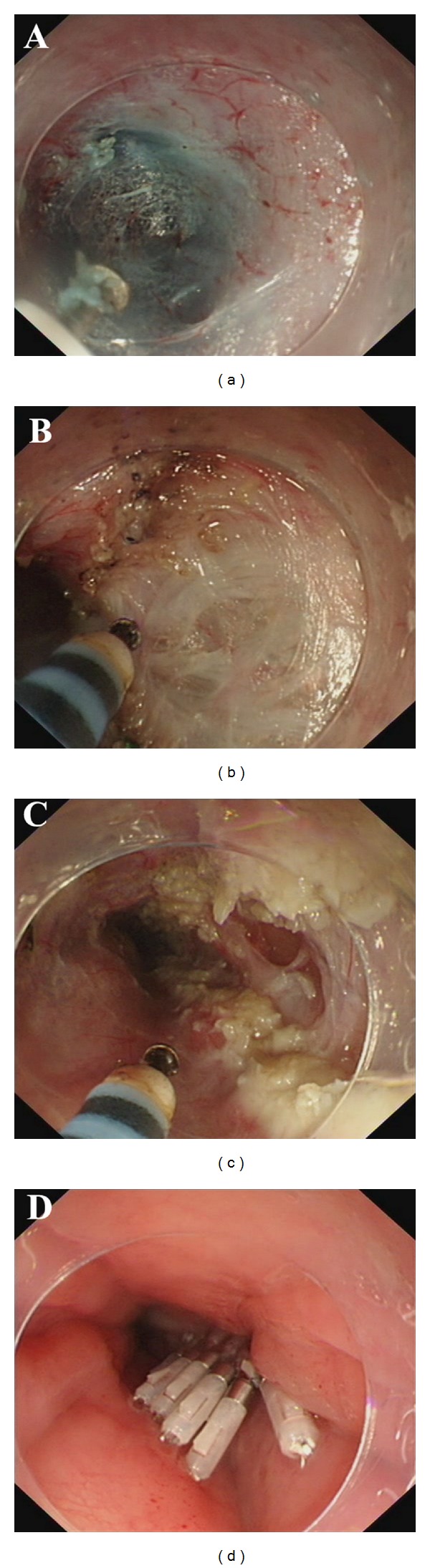
POEM procedures. (a) Creation of submucosal tunnel. A submucosal tunnel was created from the mucosal incision point (12 cm above the GEJ) to 3 cm below the GEJ. The palisade vessels on the right half of the picture confirmed the tip of the cap-fitted endoscopy had reached GEJ. The underlying inner circular muscle can also be viewed. (b) Endoscopic myotomy. The inner circular muscle was transected while remaining the outer longitudinal muscle layer intact. (c) Completion of the endoscopic myotomy. The length of myotomy was 13 cm (10 cm above the GEJ to 3 cm blow the GEJ). (d) Closure of the mucosal entry point by clips.

The patient was under general anaesthesia with intubation. The equipment and settings were presented in detail in supplementary data in supplementary material available online at doi:10.1155/2012/325479. The POEM procedure was carried out as previously described [1]. Briefly, it consisted of four steps: mucosal incision, submucosal tunnelling, myotomy, and mucosal closure (Figure 1, Videos 1–4). The main difference between Hybrid POEM and standard POEM was the use of the water-jet system. The water-jet allows for the rapid submucosal infusion of saline solution while tunnelling avoiding repeated changes of equipment. The tip of the knife was used to catch circular muscle bundles first and then to lift them up towards the tunnel for cutting. The length of myotomy was 13 cm (10 cm above the gastroesophageal junction (GEJ) to 3 cm below the GEJ). After careful hemostasis, metallic clips were applied to close the mucosal incision site. The whole procedure took 39 minutes.

The patient was on proton pump inhibitor for the prevention of reflux symptom. She was given full liquid diet on postoperative day 1. Discharged on postoperative day 2, the patient expressed an experience of “a great relief” in sternum area. During follow-up time, there was improvement in dysphagia symptom score and manometric findings (Table 1).

## 2. Discussion

 The interest in POEM has blossomed after the first report was released [1], though the golden standard for treating esophageal achalasia is still laparoscopic Heller myotomy [2]. The current case demonstrates for the first time the feasibility and the possible advantages of using HybridKnife in POEM. The most obvious advantage is shortening the operation time remarkably. As mentioned in the initial case series, the operating time ranged from 100 to 180 minutes (average 126 minutes). In this case, it only took 39 minutes. Before this case, the chief endoscopist has already performed more than 30 cases of standard POEM procedure, with an average procedure time of 76 minutes. In the standard POEM procedure, a TT knife and an injection needle were used [1]. During the creation of submucosal tunnel, the frequent change of endoscopic devices was inevitable, whereas, with the use of HybridKnife, the change of instruments was reduced. A similar conclusion was also reached with another group comparing ESD with HybridKnife and standard ESD [3]. Balloon dilation is another reported method of the creation of submucosal tunnel [4]. However, the operator cannot achieve accurate hemostasis when tunnelling by this method. Further experience is needed. A large-scale comparative study of standard POEM and Hybrid POEM is underway in our institute.

## Supplementary Material

The equipment used in the procedure included the single-channel gastroscope (GIF-H260J, Olympus), a transparent cap (D-201-11802, Olympus), haemostatic clips (HX-610-90, HX-600-135, Olympus), HybridKnife^®^ T-type (ERBE,Germany), CO2 insufflator (Olympus), the electrosurgical unit VIO^®^ 200D (ERBE, Germany, Settings: ENDO CUT Q Effect, 3, cutting width, 1, cutting interval, 4; FORCED COAG Effect, 2, Max. power 50) and the water-jet unit ERBE JET^®^ 2 (ERBE, Germany, Settings: Basic program Effect 35-40). The solution for submucosal injection was made up with 250 mL normal saline, 2.5 mL epinephrine and 2~3 mL indigo carmine.

## Figures and Tables

**Table 1:** Manometry findings and dysphagia score premyotomy and postmyotomy Month 6.

Parameters	Premyotomy	Postmyotomy
Eckardt score^∗^ (0–12)	6	0
lower esophageal sphincter (LES) pressure (mmHg)	19	8

^∗^See Table 2.

**Table 2:** Eckardt score.

Value	Dysphagia	Regurgitation	Retrosternal pain	Weight loss
0	Never	Never	Never	0 kg
1	Occasionally	Occasionally	Occasionally	0–5* *kg
2	Daily	Daily	Daily	5–10 kg
3	With every meal	With every meal	With every meal	>10 kg
