# Supplementary material for: Peroral Endoscopic Myotomy for Esophageal Achalasia by HybridKnife: A Case Report
Source: Case Rep Gastrointest Med. 2012 Jul 31;2012:325479. doi: 10.1155/2012/325479 (PMC3415097; doi:10.1155/2012/325479)
Supplement: Supplementary file 1 — The equipment used in the procedure included the single-channel gastroscope (GIF-H260J, Olympus), a transparent cap (D-201-11802, Olympus), haemostatic clips (HX-610-90, HX-600-135, Olympus), HybridKnife® T-type (ERBE,Germany), CO2 insufflator (Olympus), the electrosurgical unit VIO® 200D (ERBE, Germany, Settings: ENDO CUT Q Effect, 3, cutting width, 1, cutting interval, 4; FORCED COAG Effect, 2, Max. power 50) and the water-jet unit ERBE JET® 2 (ERBE, Germany, Settings: Basic program Effect 35-40). The solution for submucosal injection was made up with 250 mL normal saline, 2.5 mL epinephrine and 2~3 mL indigo carmine. [file 325479.f1.zip › 351616.doc]

Equipment and settings of POEM procedure

Single-channel gastroscope (GIF-H260J, Olympus)

Transparent cap (D-201-11802, Olympus)

Haemostatic clips (HX-610-90, HX-600-135, Olympus)

HybridKnife® T-type (ERBE,Germany)

CO_2_ insufflator (Olympus)

Electrosurgical unit VIO® 200D (ERBE, Germany)

Water-jet unit, ERBE JET® 2 (ERBE, Germany)

The solution for submucosal injection was made up with 250 ml normal saline, 2.5 ml epinephrine and 2~3 ml indigo carmine.

Settings of the electrosurgical unit

1. ENDO CUT Q

| Effect | 3 |
| --- | --- |
| Cutting width | 1 |
| Cutting interval | 4 |

2. FORCED COAG

| Effect | 2 |
| --- | --- |
| Max. power | 50 |

Settings of the water-jet unit (ERBE JET® 2)

Basic program

| Effect | 35~40 |
| --- | --- |
